# Inflow-weighted pulmonary perfusion: comparison between dynamic contrast-enhanced MRI versus perfusion scintigraphy in complex pulmonary circulation

**DOI:** 10.1186/1532-429X-15-21

**Published:** 2013-02-28

**Authors:** Yi-Ru Lin, Shang-Yueh Tsai, Teng-Yi Huang, Hsiao-Wen Chung, Yi-Luan Huang, Fu-Zong Wu, Chu-Chuan Lin, Nan-Jing Peng, Ming-Ting Wu

**Affiliations:** 1 Department of Electronic and Computer Engineering, National Taiwan University of Science and Technology, Taipei, Taiwan; 2 Section of Thoracic and Circulation Imaging Department of Radiology, Kaohsiung Veterans General Hospital, No.386, Ta-Chung 1st Road, 813, Kaohsiung, Taiwan, People’s Republic of China; 3 Graduate Institute of Applied Physics, National Chengchi University, Taipei, Taiwan; 4 Department of Electrical Engineering, National Taiwan University of Science and Technology, Taipei, Taiwan; 5 Department of Electrical Engineering, National Taiwan University, Taipei, Taiwan; 6 Faculty of Medicine, School of Medicine, National Yang Ming University, Taipei, Taiwan; 7 Department of Pediatrics, Kaohsiung Veterans General Hospital, Kaohsiung, Taiwan; 8 Department of Nuclear Medicine, Kaohsiung Veterans General Hospital, Kaohsiung, Taiwan; 9 Institute of Clinical Medicine, School of Medicine, National Yang Ming University, Taipei, Taiwan

**Keywords:** Pulmonary perfusion, MRI, Pulmonary scintigraphy, Dynamic contrast enhancement-MRI

## Abstract

**Background:**

Due to the different properties of the contrast agents, the lung perfusion maps as measured by 99mTc-labeled macroaggregated albumin perfusion scintigraphy (PS) are not uncommonly discrepant from those measured by dynamic contrast-enhanced MRI (DCE-MRI) using indicator-dilution analysis in complex pulmonary circulation. Since PS offers the pre-capillary perfusion of the first-pass transit, we hypothesized that an inflow-weighted perfusion model of DCE-MRI could simulate the result by PS.

**Methods:**

22 patients underwent DCE-MRI at 1.5T and also PS. Relative perfusion contributed by the left lung was calculated by PS (*PS*_*L*%_), by DCE-MRI using conventional indicator dilution theory for pulmonary blood volume (*PBV*_*L%*_) and pulmonary blood flow (*PBF*_*L%*_) and using our proposed inflow-weighted pulmonary blood volume (*PBV*^*iw*^_*L%*_). For *PBV*^*iw*^_*L%*_, the optimal upper bound of the inflow-weighted integration range was determined by correlation coefficient analysis.

**Results:**

The time-to-peak of the normal lung parenchyma was the optimal upper bound in the inflow-weighted perfusion model. Using *PS*_*L%*_ as a reference, *PBV*_*L%*_ showed error of 49.24% to −40.37% (intraclass correlation coefficient R_I_ = 0.55) and *PBF*_*L%*_ had error of 34.87% to −27.76% (R_I_ = 0.80). With the inflow-weighted model, *PBV*^*iw*^_*L%*_ had much less error of 12.28% to −11.20% (R_I_ = 0.98) from *PS*_*L%*_.

**Conclusions:**

The inflow-weighted DCE-MRI provides relative perfusion maps similar to that by PS. The discrepancy between conventional indicator-dilution and inflow-weighted analysis represents a mixed-flow component in which pathological flow such as shunting or collaterals might have participated.

## Background

Accurate assessment of pulmonary perfusion is important to the understanding of the pathophysiology of many cardiopulmonary diseases. Changes in regional lung perfusion can be observed in pulmonary diseases such as pulmonary embolism and chronic obstructive pulmonary disease [1] and sequestration [2], and in cardiovascular diseases such as pulmonary stenosis or tetralogy of Fallot (TOF) [3]. At present, the conventional method measuring regional pulmonary perfusion in clinical practice is pulmonary scintigraphy (PS) [4,5].

In recent years, MR imaging has become a competitive technique for pulmonary imaging [6-9]. MR imaging has the advantages of simultaneous acquisition of detailed anatomical images and multiple functional information to assist diagnosis without the cost of ionization radiation exposure. First-pass dynamic contrast-enhanced MR imaging (DCE-MRI) using intravenous bolus injection of contrast material has been shown to be able to detect perfusion abnormality in a semi-quantitative manner [1,6,10]. The accuracy of DCE-MRI in measuring regional pulmonary perfusion map (rPPM) has been validated by experimental studies on animal models using injected microsphere measurements as the standard [11,12]. Several studies have shown that MR imaging provides consistent rPPM compared with PS on patients with pulmonary embolism, emphysema [13] or prediction of postoperative lung function [14,15].

In patients with complex pulmonary circulation (CPC), there may be presence of pathological flow such as systemic shunting or collaterals. In these conditions, discrepancy between the rPPM by PS and DCE-MRI using the conventional dilution analysis is not uncommonly observed in clinical practice. The contrast agent used in PS, namely technetium-99m (^99m^Tc)-labeled macroaggregated albumin (MAA)(^99m^Tc-MAA), is a large aggregate with particle size on the order of some tens to about a hundred micrometers. Following intravenous injection, the ^99m^Tc-MAA gets entrapped in the pre-capillary intravascular space and temporarily obstructs approximately 1% of the total pulmonary capillary bed [16]. This capillary blockade mechanism of ^99m^Tc-MAA indicates that the PS solely provides inflow perfusion information in normal pulmonary circulation. On the contrary, the gadolinium chelate used for DCE-MRI is of several nanometers in diameter. It could thus pass freely through the capillaries to the systemic flow and re-circulation in normal lungs; or abnormally, via shunt/collateral flow to the pathological lungs such as pulmonary sequestration, which we called “mixed-flow phase” and could not be removed by gamma-variate fitting to the first-pass transit in the indicator dilution model of DCE-MRI.

Due to the presence of mixed-flow phase in DCE-MRI, we hypothesized that an inflow-weighted model of DCE-MRI could reduce discrepancy between rPPM by PS and DCE-MRI. In the present study, we developed an inflow-weighted model of DCE-MRI on 22 patients of CPC receiving both PS and DCE-MRI. We aimed to compare the inflow-weighted rPPM measured by PS and DCE-MRI in patients with CPC, which may improve the clinical utility of DCE-MRI.

## Methods

### The inflow-weighted DCE-MRI analysis

In conventional indicator dilution theory, the perfusion parameters can be derived from the series of DCE-MRI acquired throughout the first-pass contrast agent passage [17]. In particular, the relative pulmonary blood volume (rPBV) is given by the integration of the gamma-variate function-fitted first-pass signal intensity-time (SI-time) curve, *s*(*t*), after subtraction of the baseline signals [10]:

(1) $$\text{rPBV}=\int_{0}^{\infty }s\left(t\right)\text{dt}$$

Assuming that the concentration of the contrast agent is proportional to the signal enhancement, the relative mean transit time (rMTT) can be computed as the normalized first moment of the SI-time curve:

(2) $$\text{rMTT}=\frac{\int_{0}^{\infty }t\cdot s\left(t\right)\text{dt}}{\int_{0}^{\infty }s\left(t\right)\text{dt}}$$

And the relative pulmonary blood flow (rPBF) is obtained using the central volume principle:

(3) $$\text{rPBF}=\frac{\text{rPBV}}{\text{rMTT}}$$

**Figure 1:**
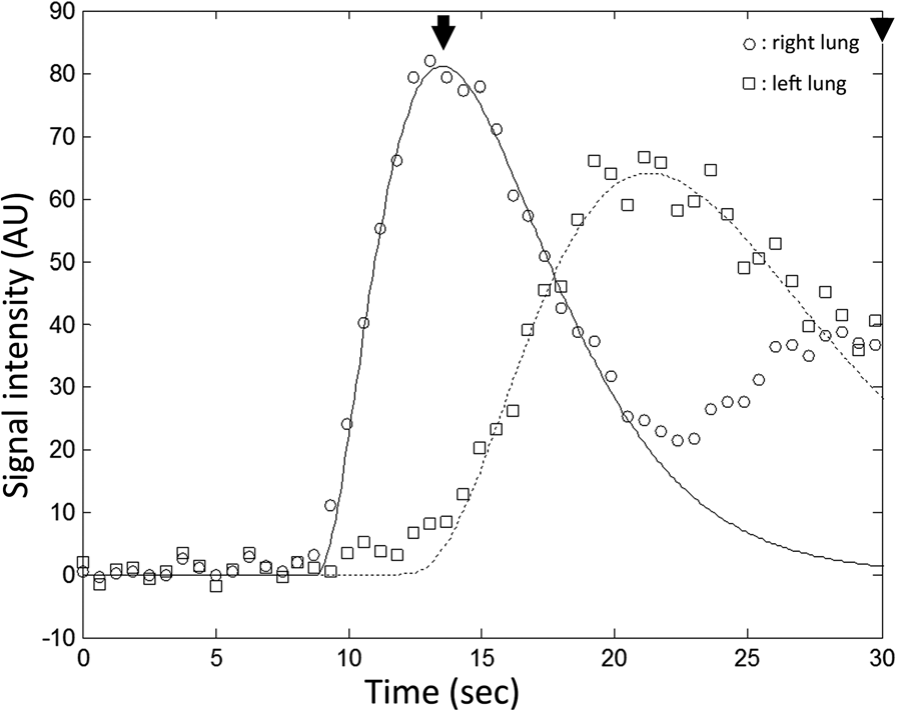
**Schematic plot of integration range for PBV and inflow-weighted analysis.** Arrowhead indicates the upper bound of integration for rPBV. The arrow indicates *t*_max_, time-to-peak for the normal lung parenchyma, which was defined as the upper bound of integration for inflow-weighted analysis.

In clinical reality, however, the conventional definition of first-pass transit may encounter difficulty in the case of CPC. In order to match the “pre-capillary” components of PS by DCE-MRI, we proposed an “inflow weighted” modification on the perfusion analysis method for DCE-MRI as follows. Instead of performing integration throughout the entire first-pass transit as in Eqs.[1]-[3], the integration range in the new method is restricted to the inflow-dominant phase. We called the new parameter rPBV^iw^; namely,

(4) $$\text{rPB}V^{\text{iw}}=\int_{0}^{t\max}s\left(t\right)\text{dt}$$

where *t*_max_, the upper bound of integration, is chosen in this study as the time where the normal lung parenchyma shows maximum signal intensity, which is often referred to as time-to-peak (Figure 1). One can see that if the integration upper bound is chosen to approach infinity instead, rPBV^iw^ would return to conventionally defined rPBV as in indicator dilution theory [10]. The rationale for choosing the time-to-peak as the integration upper bound was to ensure the wash-in portion of the pulmonary arterial phase was predominantly included, which is anticipated to give DCE-MRI results similar to those from PS. Validation of the choice of *t*_max_ is illustrated in a section below.

### Subjects

The study was approved by our institution's Internal Review Board Committee and informed consent was obtained from patients or patients' parents. The study was a second analysis of part of data which was collected in a previously published study [18]. A total of 22 patients (12 males and 10 females; age range = 3 months to 77 years, mean = 20.8 years) with CPC due to a variety of diseases without or with surgical correction were enrolled in our study. Patients with history of renal disease or impaired renal function were not enrolled. The details of patients' clinical profiles and perfusion ratios to the left lung measured by PS are shown in Table 1.

**Table 1:** Diagnostic information for patients included in this study

**Patient**	**Age**	**Sex**	**Diagnosis**	**Surgery**	*PS*_*L% *_**(%)**
1	9 y	M	TOF		97.3
2	6 y	M	TOF	Repair	0.0
3	3 y	F	TOF		33.9
4	2 y	F	Complex CHD, FSV	Hemi-Fontan	65.2
4	2 y	F	Complex CHD, FSV	Hemi-Fontan	21.2
5	4 y	M	Complex CHD, FSV	Hemi-Fontan	60.3
5	4 y	M	Complex CHD, FSV	Hemi-Fontan	1.5
6	8 y	M	Complex CHD, FSV	Fontan	82.5
6	8 y	M	Complex CHD, FSV	Fontan	13.6
7	8 y	F	Complex CHD, FSV	Fontan	68.2
7	8 y	F	Complex CHD, FSV	Fontan	1.1
8	32 y	F	Sequestration spectrum		43.7
9	3 m	F	Sequestration spectrum		29.2
10	44 y	F	Sequestration spectrum		38.4
11	61 y	F	Swyer-James		7.9
12	74 y	M	Swyer-James		68.4
13	18 m	M	TAPVR	Repair	7.3
14	17 y	M	PAPVR		98.5
15	60 y	M	Lung cancer		28.9
16	27 y	M	Pulmonary hypertension	Rt lung transplantation	30
17	77 y	M	PDA		42.2
18	12 y	M	VSD PPS	Repair	56.1
19	2 y	F	VSD	Repair	52.8
20	3 m	M	Hypogenetic lung syndrome		72.5
21	71 y	F	Pulmonary thromboembolism		32.7
22	5 m	F	Sequestration spectrum + PPS		81.6

Note – TOF: tetralogy of Fallot, CHD: congenital heart disease, FSV: functional single ventricle, TAPVR: total anomalous pulmonary venous return, PAPVR: partial anomalous pulmonary venous return, PDA: patent ductus arteriosus, VSD: ventricular septal defect, PPS: peripheral pulmonary stenosis.

*PS*_*L%*_ (%), percentage of left lung perfusion by Tc99m-MAA perfusion scintigraphy.

These patients underwent both DCE-MRI and PS to evaluate the CPC. Four patients with Fontan or bilateral Glenn’s surgery received two injections in both upper and lower limbs on two different days for perfusion scintigraphy, and with an interval of 20 minutes for DCE-MRI. The perfusion data from different limbs were analyzed and compared respectively, hence making the total number of cases 26.

### Data acquisitions

#### Lung perfusion scintigraphy

Pulmonary perfusion scintigraphy was performed after intravenous injection of ^99m^Tc-MAA. Six static views with anterior, posterior and right-left lateral oblique posterior and anterior projections were obtained with a dual-head gamma camera (Siemens E-Cam, Erlangen, Germany). In both anterior and posterior images, manually selected regions of interest (ROIs) were drawn over the left and right lungs. The percentage of perfusion of the lungs was calculated by dividing the mean radioactivity of the left lung measured from anterior and posterior views, and divided by the mean radioactivity of the whole lungs (*PS*_*L%*_).

PS_L%_ = left lung / (left lung + right lung) × 100% [5]

To obtain regional perfusion ratios, both lungs were divided to six ROIs of the upper right, middle right, lower right, upper left, middle left, and lower left lungs. The percentage perfusion ratio for each regional ROI was calculated separately (*PS*_*%*_).

#### Cardiac catheterization angiography

In 16 patients, cardiac catheterization angiography (Advantx LC/LP; GE Healthcare) was performed according to clinical indications within 3 months of the MRI study. Catheterization for the left heart and the right heart were both performed. The left ventriculography and aortography were performed first, followed by right ventriculography and pulmonary arteriography. Pressure measurements were performed at right ventricle and pulmonary trunk. Selective injections of aortic arch branches and major aortopulmonary collateral arteries were performed as needed. Selective pulmonary arteriography was performed in cases with suspected peripheral pulmonary stenosis. In cases with pulmonary atresia, wedged pulmonary venogram was performed to retrograde visualization of the pulmonary artery branches as needed.

### MR Imaging

#### Anatomical MR imaging

All MR images were acquired on a 1.5 Tesla system (GE Healthcare, Signa Cvi, Milwaukee, WI). For patients who can perform breath-holding, anatomic images were acquired with ECG-gated double inversion-recovery-prepared black-blood fast spin-echo sequence (TI/TR/TE/ETL = 340ms/1 R-R/4.7ms/32). For patients who could not hold their breath well, spin-echo T1-weighted images (TR/TE = 1 ~ 2 R-R interval/25ms) were obtained with ECG-gated and respiratory compensation during quiet breathing.

#### Dynamic contrast-enhanced MR imaging

DCE-MRI measurements were acquired by using an inversion-recovery-prepared segmented EPI technique [19] with cardiac gating. Imaging parameters, adjusted dependent on individual heart rates, were usually TI/TR/TE = 180/6.5/1.2 msec, ETL = 4, matrix size = 128×128, and interpolated to 256×256 for display. Scan slice was 6–8 mm in coronal plane. Patients were asked to hold their breath during scans as long as possible. Patients younger than 8 years were sedated and consequently were imaged during quiet breathing. For these patients, the SI-time curves showed respiration-related fluctuations which could nevertheless be smoothed via gamma-variate fitting as stated in the next section. Cardiac gating was used to avoid image misregistration, particularly for lung parenchyma near the border of mediastinum and the heart where the blood flow was relatively large and could have strong impact on pulmonary perfusion parameters. The number of slices acquired was dependent on the heart rate and about seven slices in two R-R intervals [19]. A bolus of 0.05 mmol/Kg Gd-DTPA (Magnevist, Schering, Germany) was injected intravenously after the image acquisition started, using either an MR-compatible power injector at a speed of 3 ml/sec for age > 15 years and manual injection as fast as possible for age < 15 years. A total of 40 ~ 60 frames, separated by two R-R intervals from one another, were obtained at each coronal slice locations.

#### Contrast-enhanced 3D MR angiography

The MR angiogram was acquired in coronal orientation using elliptical k-space gradient-echo imaging with TR/TE = 4.8ms/1.5ms, flip angle = 15°, matrix = 256 × 160, and field of view = 150 ~ 320 mm after the injection of Gd-DTPA (0.1 mmol/kg). Multiplanar reconstruction with maximal intensity projection was done for the interrogated vessels to evaluate the vascular morphology together with anatomical spin-echo images.

### Imaging analysis

#### Anatomical evaluation

One observer (KSH) who read the cardiac catheter angiography was blinded to the MR imaging findings and perfusion scintigraphy results with emphasis on evaluating the pulmonary arteries, aortopulmonary collaterals, and aortopulmonary shunts [20].

The anatomical axial and contrast-enhanced 3D MR angiographic imaging was evaluated with the report and imaging of catheterization angiography by two observers (YLH, MTW) in consensus. They were blinded to the results of DCE-MRI and PS.

The anatomical evaluation results were used for clinical diagnosis and to explain the possible disagreement between DCE-MRI and PS findings.

#### DCE-MRI Data analysis

An institutionally developed program on MATLAB programming environment (MathWorks, Natick, MA) was used to define the lung as ROI after excluding the heart and great hilar vessels as described previously [21]. Briefly, ROIs of both lungs were manually selected for each slice and a threshold-masking method was used to exlcude pixels with signal intensities higher than threshold. The threshold was adjusted so that the mask fit well with the anatomical borders of the lung fields. Following ROI selections, we performed baseline-intensity-subtracted and gamma-variate fittings of the SI-time curve to obtain the first-pass transit. The conventional perfusion parameters were thus derived according to Eqs.[1] to [3]. The perfusion ratios of the left lung (Eq [5]) could then be derived (*PBV*_*L%*_ and *PBF*_*L%*_). The ROIs of left and right lungs were further divided into 3 zonal ROIs, as upper, middle and lower zones, again analogous to that used in PS, in order to compare DCE-MRI with PS per person and per zone. For inflow-weighted method, rPBV^iw^ was obtained using Eq.[4] for both left lung perfusion ratio and 6-zone ROI analysis. The ROIs were identical to those in the indicator dilution method.

#### Relative pulmonary perfusion maps (rPPM)

To compare with PS, relative pulmonary perfusion maps (rPPM) of rPBV and rPBV^iw^ were calculated from DCE-MRI data. ROIs of pulmonary parenchyma were selected for each slice [21]. Analysis was then executed for every ROI in a pixel-by-pixel basis. Spatial smoothing was applied to decrease the influence of noise. rPPM was calculated from each slice and summed up as projective rPPM of whole lungs for each patient.

#### Effects of integration range for DCE-MRI

To determine the optimal integration range in Eq.[4] for rPBV^iw^ so it would be close to rPPM by PS, we developed a method to search for the optimal upper boundary of the integration range in Eq.[4], as detailed below.

We first varied the integration range in Eq.[4] by introducing a variable *ti* into the integral:

(5) $$\text{rPB}V^{\text{iw}}\left(\text{ti}\right)=\int_{0}^{t\max+\text{ti}}s\left(t\right)\text{dt}$$

where *ti* is the time interval from *t*_max_. In this study, using no more than 60 frames for each slice of DCE-MRI, *ti* ranged from −5 to 30 secs. Equivalently speaking, rPBV^iw^ is now a function of *ti*, or rPBV^iw^(*ti*). The optimal ti is the one where rPBV^iw^(*ti*) is most similar to rPPM by PS. To find this optimal *ti*, the DCE-MRI data were first analyzed to yield rPBV^iw^(*ti*), from which the corresponding *PBV*^*tw*^_*L%*_*(ti*) values were derived. These *PBV*^*tw*^_*L%*_*(ti*) values were compared with the nuclear medicine data *PS*_*L%*_. In order to assess the overall agreement for the 26 cases, we calculated the correlation coefficient between *PBV*^*tw*^_*L%*_*(ti)* and *PS*_*L%*_ for each *ti*. Consequently, the optimal *ti* value for *PBV*^*tw*^_*L%*_*(ti)* was chosen as the one that yielded the largest correlation coefficient, and was used for all regional inflow-weighted analysis as stated in previous sections.

### Statistical analysis

The overall agreement between PS and DCE-MRI was assessed using the intraclass correlation coefficient [22], which produces measures of consistency or agreement of values within cases. In addition, graphical visualization of the agreement between perfusion ratios of PS and DCE-MRI was assessed using the Bland-Altman analysis [23].

## Results

**Figure 2:**
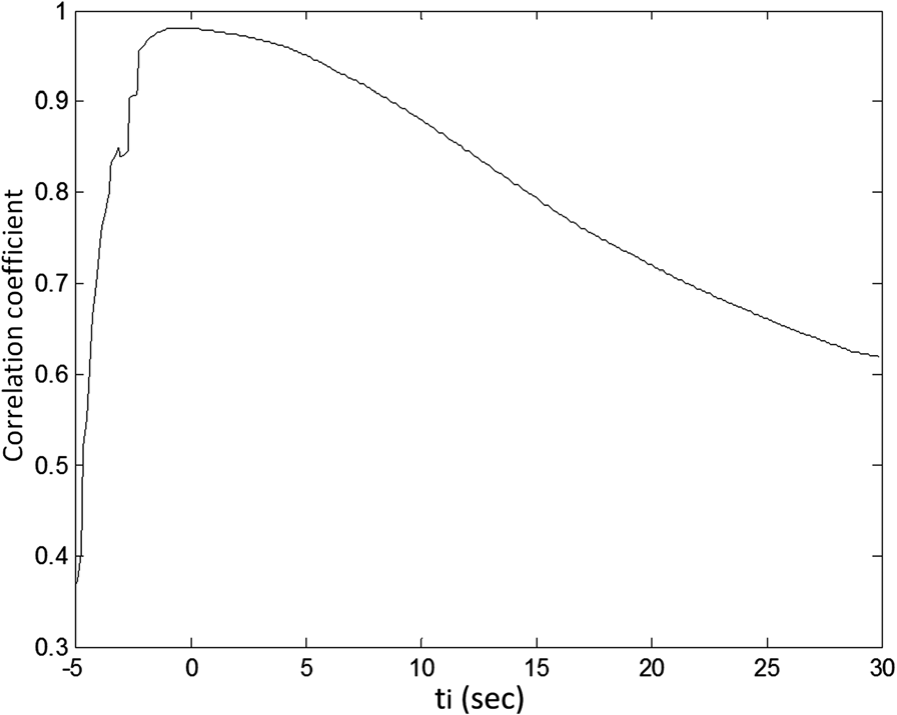
**Influence of integration range *ti *on rPBV**^**iw**^**.** Correlation coefficient between *PBV*^*iw*^_*L%*_(*ti*) and *PS*_*L%*_ reached maximum value of 0.981 at *ti −*0.3sec, which is almost the time-to-peak (*ti* =0). We used time-to-peak *t*_max_ as the upper bound for inflow-weighted analysis of DCE-MRI.

Figure 2 shows the effects of integration range used in inflow-weighted DCE-MRI. The choice of the integration upper bound greatly influenced the calculated perfusion ratio. It is seen that the integration range corresponding to the largest correlation coefficient of 0.98 is very close to the time-to-peak (*ti* = −0.3 sec). For computational convenience, the time-to-peak *t*_max_ was therefore chosen as the optimal integration upper bound for *PBV*^*tw*^_*L%*_*(ti)*.

**Figure 3:**
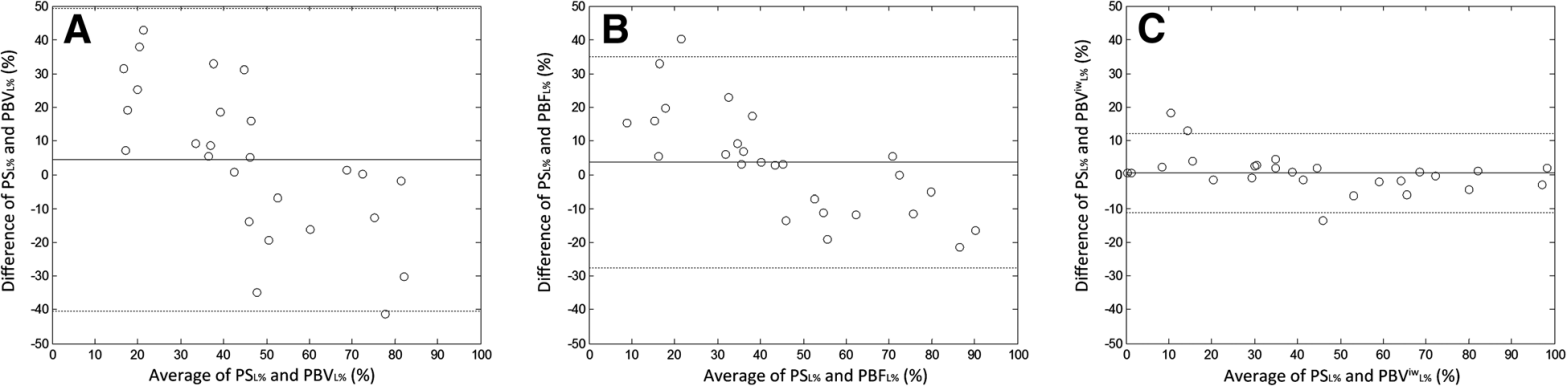
**Bland-Altman analysis plots of the percent flow to the left lung compared with PS, (A) rPBV (B) rPBF (C) rPBV**^**iw **^**analysis.** rPBV^iw^ shows the best consistency with the PS. PS, perfusion scintigraphy, rPBV, relative pulmonary blood volume, rPBF, relative pulmonary blood flow, rPBV^iw^, inflow-weighted relative pulmonary blood volume.

Table 2 lists the perfusion parameters including rPBV, rPBF, and rPBViw in both left and right lungs, and the corresponding percentage of perfusion of the lungs. The Bland-Altman plots [23] in Figure 3A and B show the overall agreement between *PS*_*L%*_ and *PBV*_*L%*_ or *PBF*_*L%*_ respectively. For rPBV versus PS (Figure 3A), the mean difference was 4.43%, with upper and lower limits of two standard deviations at 49.24% and −40.37% respectively. The intraclass correlation coefficient [22] between these two methods was R_I_ = 0.55 (95% confidence interval [0.22, 0.77]). For rPBF versus PS (Figure 3B), the mean difference was 3.55% (upper and lower limits at 34.87% and −27.76% respectively), with intraclass correlation coefficient R_I_ = 0.80 and 95% confidence interval [0.60, 0.90]. These results suggest that both rPBV and rPBF showed substantial discrepancy with PS in our subjects with CPC. With the inflow-weighted model, *PBV*^*tw*^_*L%*_ (Figure 3C), the range of *PBV*^*iw*^_*L%*_ − *PS*_*L%*_ discrepancy was 12.28% and −11.20% respectively. Mean difference was 0.54% and R_I_ = 0.98 (95% confidence interval [0.96, 0.99]).

**Table 2:** Perfusion parameters including rPBV, rPBF, and rPBViw in both left and right lungs

**Patient**	**rPBV (A.U.)**		**PBV**_**L% **_**(%)**	**rPBF(A.U.)**		**PBF**_**L% **_**(%)**	**rPBV**^**iw **^**(A.U.)**		**PBV**^**iw**^_**L% **_**(%)**
	**Left**	**Right**		**Left**	**Right**		**Left**	**Right**	
1	1587157	136884	92.1	6011058	4782211	55.7	609401	266672	69.6
2	4184	789734	0.5	1809734	2426058	42.7	174724	346700	33.5
3	1719347	3091112	35.7	6218989	9614279	39.3	776058	1315645	37.1
4	753700	438674	63.2	2488098	5723882	30.3	248556	289924	46.2
4	216036	881601	19.7	4039850	3424204	54.1	312637	395298	44.2
5	1995797	1441262	58.1	7676676	11091451	40.9	398871	415493	49.0
5	917936	3760608	19.6	7653578	11810187	39.3	492393	688432	41.7
6	1536567	438084	77.8	4343320	1043287	80.6	569178	166541	77.4
6	588633	2753401	17.6	1892854	7243710	20.7	137425	582693	19.1
7	1174442	527101	69.0	3635513	1593516	69.5	466754	168287	73.5
7	7572	475954	1.6	575611	1198385	32.4	67059	339961	16.5
8	951568	1138064	45.5	3028626	3167695	48.9	301882	343895	46.7
9	247186	525474	32.0	2329719	1522402	60.5	303914	345572	46.8
10	431442	671381	39.1	2247450	1892474	54.3	192487	265560	42.0
11	233023	876317	21.0	1139290	3049546	27.2	68795	179289	27.7
12	822685	484056	63.0	2352025	2152277	52.2	199549	153775	56.5
13	178215	1726389	9.4	2506232	5197082	32.5	461815	1515113	23.4
14	1047594	49729	95.5	2775160	2076806	57.2	434155	95379	82.0
15	589305	1288370	31.4	2272678	3708367	38.0	188174	348941	35.0
16	609832	1500893	28.9	3780830	3990400	48.7	253484	394298	39.1
17	357424	524454	40.5	1111662	1479468	42.9	99362	121789	44.9
18	736335	739105	49.9	2415417	2495677	49.2	339765	353479	49.0
19	1297335	2001479	39.3	3852779	6041393	38.9	665898	1033059	39.2
20	2784430	1080745	72.0	8378116	3174017	72.5	1080888	410446	72.5
21	363549	612778	37.2	1507310	2132760	41.4	165563	253541	39.5
22	774967	162284	82.7	2305914	1037507	69.0	473009	202386	70.0

A.U. = arbitrary unit.

**Figure 4:**
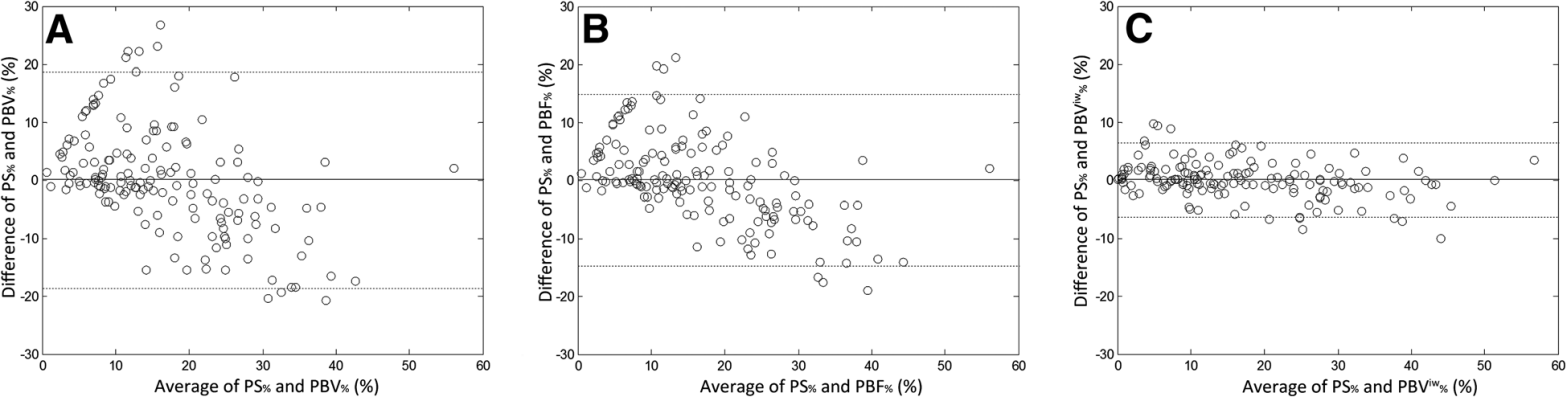
**Bland-Altman analysis plots of the percent flow to 6 ROIs compared with PS, (A) rPBV (B) rPBF (C) rPBV**^**iw **^**analysis.** rPBV^iw^ shows the best consistency with PS.

The 6-zonal correlative study to *PS*_*%*_ also showed similar improvement in inflow-weighted analysis. Zonal *PBV*_*%*_ and *PBF*_*%*_ are plotted versus *PS*_*%*_ in Figure 4A and B respectively. Figure 4C shows *PBV*^*tw*^_*%*_ with integration upper boundary chosen at the point of time-to-peak.

**Figure 5:**
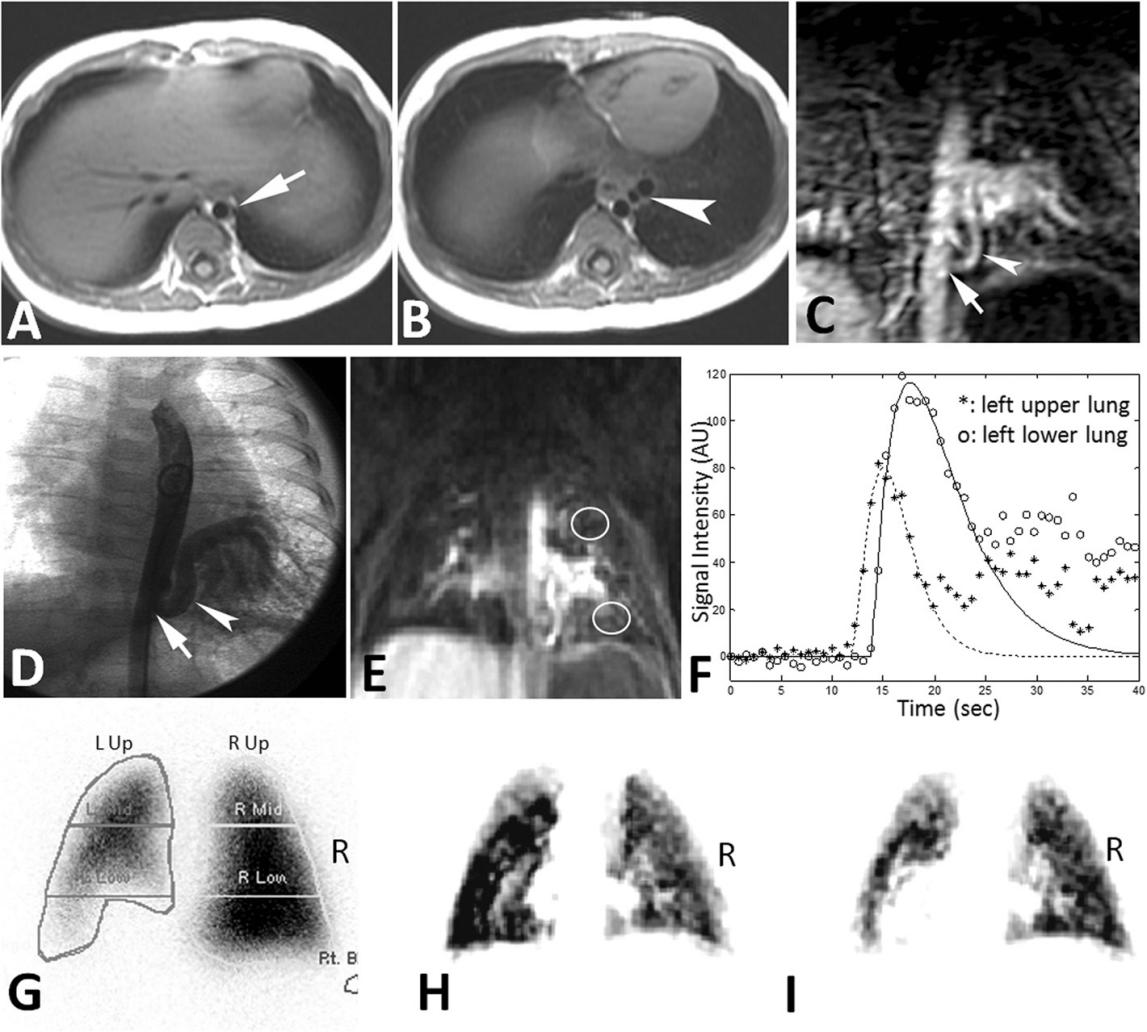
**A case of a 3-month-old female with pulmonary sequestration spectrum.** (**A, B**) Axial ECG-gated spin-echo T1WI at diaphragm level (**A**): an anomalous arterial supply from descending aorta (arrow). (**B**), the artery courses serpiginously (arrowhead) supplying the basal segments of the left lower lung. (**C**) Contrast-enhanced 3D MR angiography: An anomalous arterial supply from aorta (arrow) to the left lower lung (arrowhead). The course and branching pattern in the basal segments was identical to those confirmed by catheter angiography (**D**). (**E**) DCE-MRI after bolus injection. Two ROIs were chosen on left upper lung and left lower lung. (**F**) SI-time curve of left low lung showed a delayed bolus-arrival as compared to curve of left upper lung. (**G**) Tc-99M-MAA pulmonary perfusion scintigraphy (posterior-anterior view) shows decreased perfusion over the left lower zone. (**H**) rPBV map of whole lungs calculated from DCE-MRI. There was no obvious flow deficit in rPBV map. (**I**) rPBViw map of the whole lungs. It is similar to Figure (**H**) except that inflow-weighted analysis was calculated instead of rPBV analysis. rPBViw map showed a flow deficit in the left lower lung.

**Figure 6:**
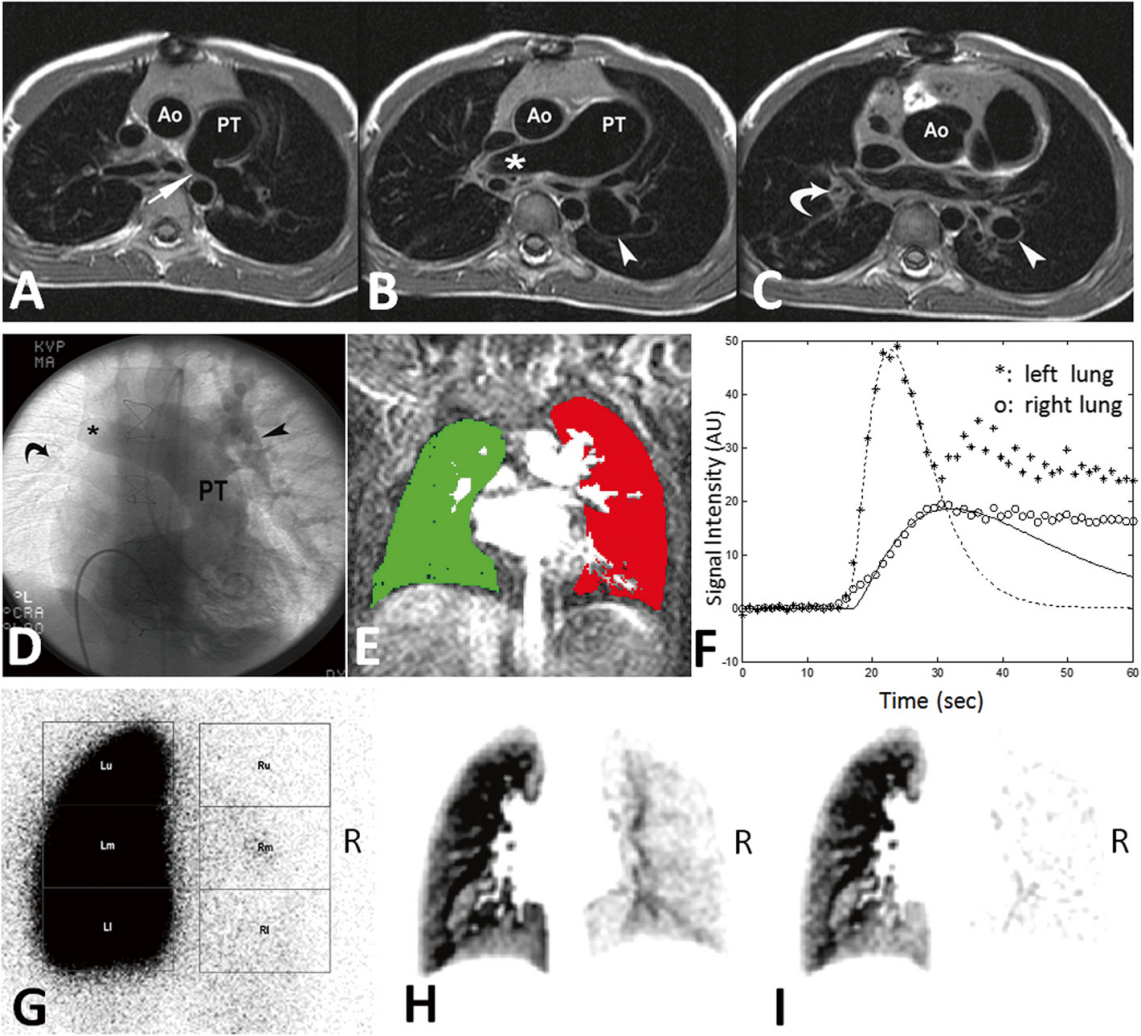
**(A-C) A serial of ECG-gated spin echo T1-weighted image.** At the carinal level (**A**), a moderate degree of stenosis (arrow) of left pulmonary artery was noted. At right pulmonary artery level (**B**), the dilated right pulmonary artery shows rapid tapering (asterisk). Note the left pulmonary artery shows post-stenotic dilatation (arrow). At the lower lung level (**C**), the right pulmonary artery is tiny (curved arrow), in contrast to the left lower pulmonary artery (arrowhead). Ao: aorta; PT: pulmonary trunk. (**D**) Catheter angiography with contrast medium injection from right ventricle shows dilated PT, left pulmonary artery (arrowhead) and right pulmonary artery with an abrupt tapering (asterisk). Note there is a tiny branch with decrease flow (curved arrow) in the right lower lung. (**E**). DCE-MRI after bolus injection. ROIs were chosen to cover the whole left lung (red) and right lung (green). Pixels with high intensity were considered as vessels and excluded from ROIs. (**F**) SI-time curves obtained from DCE-MRI for the left and right lungs. (**G**) Tc99M-MAA pulmonary perfusion scintigraphy (posterior-anterior view) shows almost no detectable perfusion in the right lung. (Right, 97.3%, Left, 2.7%). (**H**) rPBV map of whole lungs showed a small but observable flow in the right lung. (**I**) rPBViw map of the whole lungs was similar to PS and showed no detectable flow in the right lung.

Example cases in Figure 5 show a 3-month-old girl with lung sequestration spectrum. Figure 6 shows a 9-year-old boy with repaired TOF with bilateral peripheral pulmonary stenosis. The rPBV^iw^ maps (Figures 5I, 6I) had better agreement with the PS perfusion map in Figures 5G and 6G respectively.

## Discussion

This is the first study to systematically investigate the discrepancy between rPPM by DCE-MRI and PS. We showed that the rPPM by inflow-weighted DCE-MRI was very close to PS, as compared to the indicator dilution DCE-MRI. The discrepancy between the indicator dilution DCE-MRI and PS (and analogically, between indicator dilution vs. inflow-weighted DCE-MRI) was mixed-flow components (which mixed with normal re-circulation and abnormal shunting or collateral flow) after the first pass perfusion. Our result suggests that we could identify the inflow-weighted component of DCE-MRI, which might improve DCE-MRI for comprehensive evaluation of CPC.

There are great differences in particle sizes of DTPA and MAA. Normally, MAA particles (around 10–100 micrometer in size) are much larger than red blood cells (7 micrometer) and cannot pass through the capillaries normally, while DTPA particles are much smaller (<10 nanometer in size) and continually flow to the left heart system, travel back to the right heart system and re-circulate to the pulmonary artery as secondary pass. In terms of hemodynamic pathway, PS purely represents the inflow component of the first pass transit of pulmonary perfusion, while DCE-MRI contains inflow-weighted and mixed-flow phases contaminated by normal re-circulation or abnormal shunting or collateral flow.

In previous investigations comparing rPPM by dilution-model DCE-MRI (rPBF) vs. PS in human subjects, the inter-modality correlation has been reported as generally good (r = 0.84 ~ 0.92) [1,13-15,24]. Taking an in-depth look, large discrepancies between rPPM by PS and dilution model DCE-MRI have been observed, but neglected, in certain cases of these studies. For example, one-third of the patients with malignant stenosis of the pulmonary artery showed about 40% discrepancy in rPPM between PS and DCE-MRI [25]; also reported in patients with a variety of lung diseases [26]. All the evidence [25,26] together with the present study indicates that rPPM by PS and by dilution method DCE-MRI is not equivalent in all pulmonary diseases.

In DCE-MRI, “wash-out” effect actually starts during the up-slope part in a much less magnitude than the “wash-in” effect; therefore, we used the term “inflow-weighted” DCE-MRI in comparison with PS. As shown in Figure 2, the optimal cut-off point to represent the inflow-weighted part was the curve peak of the control lung parenchyma. According to our previous study on bolus tracking in CPC [18], at this particular time point, the front end of the contrast bolus had almost arrived at the left heart. Therefore, the systemic collateral flow due to aberrant artery (as Figure 5) or stenotic pulmonary artery (as Figure 6) would largely be excluded in the inflow-weighted analysis. We think this is the hemodynamic foundation of rPBV^iw^ that could identify inflow-weighted component of CPC.

It is reasonable to speculate that subtraction of rPBV by rPBV^iw^ is equivalent to the mixed-flow phase of rPBV. The mixed-flow phase of rPBV might be a meaningful indicator in CPC. With integrated usage of the temporal correlation technique for bolus tracking visualization [18], differential assessment of inflow-weighted phase vs. mixed-flow phases of rPPM is potentially of great impact on the pathophysiological evaluation of CPC.

One may propose a variation of inflow-weighted DCE-MRI; e.g. integration of area-under-curve to the peak of normal lung and the peak of abnormal lung separately. In fact, we have tested it and found the results were similar to that by the conventional indicator-dilution model (rPBV and rPBF).

The rPBV and rPBV^iw^ by DCE-MR are relative, not absolute measurements. We found a very high inter- and intra-rater variability in the measurement of arterial input function; therefore, we did not apply AIF correction to obtain absolute PBV. In addition, we cannot calculate rPBF^iw^ since we cannot estimate the mean transit time for inflow.

There was perfusion defection around the hilar region in rPPM of rPBV or rPBV^iw^, as compared to PS (Figures 5G-I, 6G-I). This was owing to the partial volume effect of the large hilar vessels which were over-sized masked by signal intensity-threshold method during ROI selection of lung parenchyma. Although most studies use one or two slices only for rPBV or rPBF [10,11], we believe application of faster scan technique to improve the scan coverage and slice thickness would solve this phenomenon.

This study design had limitations as the subjects comprised a wide variety of disease entities and a wide range of ages, in retrospective analysis. A prospective study on a specific disease entity such as Fontan physiology incorporated with more hemodynamic information in additional to PS would further validate the clinical impact of our proposed method.

## Conclusions

In conclusion, the present study supported our hypothesis that discrepancies of rPPM between PS and the indicator-dilution model of DCE-MRI in CPC could be improved by the implantation of the inflow-weighted model of DCE-MRI. DCE-MR can identify inflow-weighted and mixed-flow phases in the full spectrum of pulmonary perfusion; therefore, it could be a substitute for PS in clinical practice. We suggest that, in patients with complex cardiopulmonary disease, the conventional indicator-dilution model and our inflow-weighted model of DCE-MRI should both be integrated for comprehensive evaluation of CPC.

## Abbreviations

TOF: Tetralogy of Fallot; PS: Perfusion scintigraphy; DCE-MRI: Dynamic contrast enhanced MRI; rPPM: Regional pulmonary perfusion map; CPC: Complex pulmonary circulation; 99mTc-MAA: Technetium-99m -labeled macroaggregated albumin; rPBV: Relative pulmonary blood volume; rMTT: Relative mean transit time; rPBF: Relative pulmonary blood flow; SI-time: Signal intensity-time; ROI: Region of interest. Presented in part at the Twelfth Annual Meeting of the International Society for Magnetic Resonance in Medicine.

## Competing interests

No competing interests.

## Authors’ contributions

YRL participated in the design of the study and the proportion of analysis algorithm, carried out MRI data analysis and drafted the manuscript. SYT participated in the proportion of analysis algorithm and carried out MRI data analysis and manuscript revision. TYH participated in the proportion of analysis algorithm and manuscript revision. HWC participated in the proportion of analysis algorithm and helped to draft the manuscript. YLH participated in data interpretation and manuscript revision. FZW participated in data interpretation and manuscript revision. CCL participated in data acquisition/analysis and manuscript revision. NJP participated in data acquisition/analysis and manuscript revision. MTW participated in the design of the study, evaluated angiographic imaging, performed statistical analysis and manuscript editing. All authors read and approved the final manuscript.Grant sponsor: National Science Council of Taiwan, Grant number: NSC96-2628-E-002-006-MY3, NSC98-2221-E-011-094, NSC100-2314-B-010 -045 -MY3.
